# Supplementary material for: Extraction of active RhoGTPases by RhoGDI regulates spatiotemporal patterning of RhoGTPases
Source: eLife. 2019 Oct 24;8:e50471. doi: 10.7554/eLife.50471 (PMC6910828; doi:10.7554/eLife.50471)
Supplement: Supplementary file 1. [file elife-50471-supp1.docx]

| RhoGTPase:nucleotide | WT RhoGDI  orthologue | Apparent affinity  (μM) | Maximum velocities (sec^-1^) |
| --- | --- | --- | --- |
| Cdc42:GDP | *X. laevis* | 3.41±0.56 | 0.51±0.02 |
| Cdc42:GDP | *B. taurus* | 2.20±0.58 | 0.41±0.03 |
| Cdc42G12V:GTPγS | *X. laevis* | 4.95±1.07 | 0.35±0.03 |
| Cdc42Q61L:GTP | *X. laevis* | 14.39±2.50 | 0.19±0.02 |
| Cdc42Q61L:GTP | *B. taurus* | 2.23±0.17 | 0.28±0.01 |
| Rho:GDP | *X. laevis* | 5.77±0.87 | 0.65±0.03 |
| Rho:GDP | *B. taurus* | 2.68±0.30 | 0.84±0.05 |
| RhoG14V:GTPγS | *X. laevis* | 5.43±1.70 | 0.36±0.05 |
| RhoQ63L:GTP | *X. laevis* | 19.58±2.45 | 0.33±0.02 |
| RhoQ63L:GTP | *B. taurus* | 1.66±0.25 | 0.32±0.03 |

| RhoGTPase:nucleotide | QQ RhoGDI  orthologue | Apparent affinity  (μM) | Maximum velocities (sec^-1^) |
| --- | --- | --- | --- |
| Cdc42:GDP | *X. laevis* | 5.70±1.31 | 0.41±0.03 |
| Cdc42:GDP | *B. taurus* | 1.99±0.43 | 0.26±0.02 |
| Cdc42G12V:GTPγS | *X. laevis* | 0.94±1.02 | 0.02±0.01 |
| Cdc42Q61L:GTP | *X. laevis* | 36.76±58.69 | 0.02±0.03 |
| Cdc42Q61L:GTP | *B. taurus* | 3.84±0.54 | 0.04±0.003 |
| Rho:GDP | *X. laevis* | 15.50±2.63 | 0.57±0.05 |
| Rho:GDP | *B. taurus* | 0.89±0.07 | 0.36±0.05 |
| RhoG14V:GTPγS | *X. laevis* | 6.64±0.59 | 0.23±0.01 |
| RhoQ63L:GTP | *X. laevis* | NM* | NM* |
| RhoQ63L:GTP | *B. taurus* | 7.72±3.00 | 0.16±0.05 |

*No meaningful fit was possible
