# Supplementary material for: Extraction of active RhoGTPases by RhoGDI regulates spatiotemporal patterning of RhoGTPases
Source: eLife. 2019 Oct 24;8:e50471. doi: 10.7554/eLife.50471 (PMC6910828; doi:10.7554/eLife.50471)
Supplement: Supplementary file 2. [file elife-50471-supp2.docx]

| Primer Name | Primer sequence (5’-3’) |
| --- | --- |
| Cdc42(1) | cccgggcccgggaattcatgcagacaattaaatggtgtagtcgttgg |
| Cdc42(2) | cgcgccagagcatgcggatccgccagactgtttgttttttgccag |
| Cdc42(3) | gatccgcatgctctggcgcgccgggcaaaccaatc |
| Cdc42(4) | cccgcgcggccctcgagttatagcagcatacacttgcgtttcttc |
| Cdc42(Q61L)fwd | ctttttgatactgcagggctagaggattatgacagattacg |
| Cdc42(Q61L)rev | cgtaatctgtcataatcctctagccctgcagtatcaaaaag |
| Cdc42(G12V)fwd | gtgttgttgtgggcgatgttgctgttggtaaaacatg |
| Cdc42(G12V)rev | catgttttaccaacagcaacatcgcccacaacaacac |
| GTPase(GeneStrand)fwd | gagaatctttattttcagggc |
| GTPase(GeneStrand)rev | ggtggtggtgctcgagtgc |
| Cdc42(Sf9)fwd | ccactactgagaatctttattttcagggtggtggtggtggtatgcagacaattaagtgtgttgttg |
| Cdc42(Sf9)rev | gcaggctctagattcgaaagcgttattatagcagcacacacctgcgg |
| pETfwd | ggccgcactcgagcaccacc |
| pETrev | cgccctgaaaataaagattctc |
| pFASTBacH10fwd | cgctttcgaatctagagcctgc |
| pFASTBacH10rev | ctgaaaataaagattctcagtagtgg |
| pCS2+-Cdc42(1) | cttgttctttttgcaggaaaccatcgattcgaattcatgcagac |
| pCS2+-Cdc42(2) | tcgaatcgatggtttcctgcaaaaagaacaagtagcttgtattc |
| Rac(1) | cccgggcccggaattcatgcaggccattaaatgtgtgg |
| Rac(2) | cgcgccagagcatgcggatccgccagagagtttcttttctttcagcttctcaatagtgtc |
| Rac(3) | gatccgcatgctctggcgcgccgggcacccc |
| Rac(4) | cccgcgcggccctcgagttacaacagccgacatcttc |
| pCS2+-Rac(1) | ctttttgcaggcccccatcgatatgcagccattaaatgtg |
| pCS2+-Rac(2) | gcctgcatatcgatgggggcctgcaaaaagaacaagtagc |
| Rho(1) | cccgggcccggaattcatggcagccattcgtaagaagctcg |
| Rho(2) | cgcgccagagcatgcggatccgccagactgtttcattttggtgagctccct |
| Rho(3) | gatccgcatgctctggcgcgccgggcgagcctgtgaag |
| Rho(4) | cccgcgcggccctcgagttagatgagaaggcacgtgg |
| Rho(Q63L)fwd | gggacacagctgggctggaagattatgatcgc |
| Rho(Q63L)rev | gcgatcataatcttccagcccagctgtgtccc |
| Rho(G14V)fwd | atgtctttccacaggctacatcaccaacaatcacc |
| Rho(G14V)rev | ggtgattgttggtgatgtagcctgtggaaagacat |
| Rho(N41V)fwd | ccagaagtgtatgtcccaacagtttttgaggtctatgtggcagac |
| Rho(N41V)rev | cttgccatccacttctatgtctgccacatagacctcaaaaactg |
| Rho(Sf9)fwd | ccactactgagaatctttattttcagggtggtggtggtggtatggctgccatccggaagaaac |
| Rho(Sf9)rev | gcaggctctagattcgaaagcgttattacaagacaaggcaaccagattttttc |
| pCS2+-Rho(1) | caagctacttgttctttttgcaggatggcatcgattcgaattc |
| pCS2+-Rho(2) | ggctgccatgaattcgaatcgatgccatcctgcaaaaagaac |
| eGFP(1) | ctggcggatccatggtgagcaaggg |
| eGFP(2) | gcattggcgcgcccttgtacagctc |
| 8(A)F1 | ggcgctgcagctgtggcagcagcagtcgaactgaac |
| 8(A)F2 | ggccgcaaaggcaggcatcaagcatggcgctgcagctg |
| 8(A)F3 | gatcccatcgattcgaattcatggccgcaaaggcag |
| 8(A)F4 | cgagctgtacaagtccggaatggccgcaaaggcag |
| R1 | cactatagttctagaggctcgagttaatctttccactctttc |
| R2 | gaattcgaagcttgagctcgacgttaatctttccactc |
| HR F1 | ggacaagggaggtggtggaagtggtggaggaggttctgcccaagtggatcc |
| HR F2 | tgaactacaaggccccggagatgaaatctctgcaggaaatccaagagttggacaagggag |
| HR F3 | catcaagcatggcgaggaggaggtggaagaagaagtcgaactgaactacaag |
| HR F4 | gatcccatcgattcgaattcatggccgacaaggatggcatcaagcatgg |
| HR F5 | cgagctgtacaagtccggaatggccgacaaggatggcatcaagcatgg |
| (-)20 F1 | ggatcccatcgattcgaattcatgaactacaaggccccg |
| (-)20 F2 | gctgtacaagtccggatgaactacaaggcccc |
| (-)55 F1 | gatcccatcgattcgaattcatggcccaagtggatcctaaccttc |
| (-)55 F2 | cgagctgtacaagtccggaatggcccaagtggatcctaaccttc |
| XlRhoGDIfwd | ctgttccaggggcccctgggatcctgtatggccgacaaggatggc |
| XlRhoGDIrev | gatcgtcagtcagtcacgatgcggccgcttatcaatctttccactctttctttatgg |
| E163/4Qfwd | gtatgagttcctgacccccatgcagcaggcgcccaagggcatgc |
| E163/4Qrev | gcatgcccttgggcgcctgctgcatgggggtcaggaactcatac |
| Δ1-22fwd | ctgttccaggggcccctgggatcctgtcactcagtcaactataagc |
| Δ1-22rev | cgtcagtcagtcacgatgcggccgctcagtccttcc |
| Δ1-59fwd | ctgttccaggggcccctgggatcctgtgctgtgtctgctgacccc |
| Δ1-59rev | cgtcagtcagtcacgatgcggccgctcagtccttcc |
| HRfwd | gtggtggaagtggtggaggaggttctgctgtgtctgctgacccc |
| HRrev | ctcctccaccacttccaccacctcccttatagttgactgagtgctcg |
| pGEXHRfwd | cgagcactcagtcaactataagggaggtggtggaagtggtggaggaggttctgctgtgtctgctgacccc |
| pGEXHRrev | cgagcactcagtcaactataagggaggtggtggaagtggtggaggag |
